# Exploring the pharmacological mechanism of *Glycyrrhiza uralensis* against KOA through integrating network pharmacology and experimental assessment

**DOI:** 10.1111/jcmm.18319

**Published:** 2024-05-14

**Authors:** Jianbo Xu, Qi Sun, Min Qiu, Yungang Wu, Liangyan Cheng, Nanwan Jiang, Ruogu Zhang, Jiali Chen, Wenhua Yuan, Hongting Jin, Weidong Wang, Yunhuo Cai, Chunchun Zhang, Pinger Wang

**Affiliations:** ^1^ Institute of Orthopedics and Traumatology The First Affiliated Hospital of Zhejiang Chinese Medical University (Zhejiang Provincial Hospital of Chinese Medicine) Hangzhou China; ^2^ College of Pharmaceutical Sciences Zhejiang Chinese Medical University Hangzhou China; ^3^ The First People's Hospital of Xiaoshan District Xiaoshan Affiliated Hospital of Wenzhou Medical University Wenzhou China; ^4^ Department of Orthopedic Joint Surgery Hangzhou Fuyang Hospital of TCM Orthopaedics and Traumatology Hangzhou China; ^5^ Department of the Orthopedics of TCM The First Affiliated Hospital of Wenzhou Medical University Wenzhou China; ^6^ Hangzhou Yiyuan Pharmaceutical Technology Co., Ltd. Hangzhou China; ^7^ The First College of Clinical Medicine Zhejiang Chinese Medical University Hangzhou China; ^8^ Department of the Orthopedic Surgery The Second Affiliated Hospital of Zhejiang Chinese Medical University Hangzhou China; ^9^ Department of the Orthopedic Surgery The Third Affiliated Hospital of Zhejiang Chinese Medical University Hangzhou China

**Keywords:** *Glycyrrhiza uralensis* Fisch., inflammatory response, knee osteoarthritis, molecular docking, network pharmacology

## Abstract

Knee osteoarthritis (KOA), a major health and economic problem facing older adults worldwide, is a degenerative joint disease. *Glycyrrhiza uralensis* Fisch. (GC) plays an integral role in many classic Chinese medicine prescriptions for treating knee osteoarthritis. Still, the role of GC in treating KOA is unclear. To explore the pharmacological mechanism of GC against KOA, UPLC‐Q‐TOF/MS was conducted to detect the main compounds in GC. The therapeutic effect of GC on DMM‐induced osteoarthritic mice was assessed by histomorphology, μCT, behavioural tests, and immunohistochemical staining. Network pharmacology and molecular docking were used to predict the potential targets of GC against KOA. The predicted results were verified by immunohistochemical staining Animal experiments showed that GC had a protective effect on DMM‐induced KOA, mainly in the improvement of movement disorders, subchondral bone sclerosis and cartilage damage. A variety of flavonoids and triterpenoids were detected in GC via UPLC‐Q‐TOF/MS, such as Naringenin. Seven core targets (JUN, MAPK3, MAPK1, AKT1, TP53, RELA and STAT3) and three main pathways (IL‐17, NF‐κB and TNF signalling pathways) were discovered through network pharmacology analysis that closely related to inflammatory response. Interestingly, molecular docking results showed that the active ingredient Naringenin had a good binding effect on anti‐inflammatory‐related proteins. In the verification experiment, after the intervention of GC, the expression levels of pp65 and F4/80 inflammatory indicators in the knee joint of KOA model mice were significantly downregulated. GC could improve the inflammatory environment in DMM‐induced osteoarthritic mice thus alleviating the physiological structure and dysfunction of the knee joint. GC might play an important role in the treatment of knee osteoarthritis.

## 1 INTRODUCTION

Knee osteoarthritis (KOA), a major health and economic problem facing older adults worldwide, is a degenerative joint disease characterized by progressive degeneration of articular cartilage and inflammation of the synovium.^1^, ^2^ Damaged or diseased joints can cause severe pain and limited mobility, which impairs the life quality of the afflicted individual.^3^ However, the pathogenesis of KOA is complex and involves multiple factors, including mechanical stress, obesity, ageing, genetic inheritance and metabolic changes.^4^, ^5^, ^6^, ^7^ Inflammatory environment leads to progressive inflammation of the joints and destruction of articular cartilage and bone. Studies have shown that 95% of the hyaline cartilage in the knee joint is composed of extracellular matrix, which provides a stable structural basis for the integrity of the cartilage. Nevertheless, pro‐inflammatory cytokines such as interleukin (IL)‐1β drive the production of catabolic enzymes that lead to the breakdown of the extracellular matrix in osteoarthritic cartilage.^8^, ^9^, ^10^

*Glycyrrhiza uralensis* Fisch. (GC) is a herbaceous perennial legume copiously cultivated in many places worldwide, more commonly used for detoxification and anti‐inflammatory.^11^, ^12^, ^13^ GC contains a variety of bioactive components, mainly triterpenoids and flavonoids.^14^, ^15^ In clinical settings, GC is widely used for its anti‐inflammatory and anti‐fungal properties. Previous experiments have shown that GC reduces the expression of inflammatory cytokines such as tumour necrosis factor‐α (TNF‐α), interleukin‐1 (IL‐1) and interleukin‐6 (IL‐6).^16^, ^17^ Additionally, GC plays an integral role in many classic Chinese medicine prescriptions for treating knee osteoarthritis, such as Duhuo Jisheng formula and Bushen Huoxue decoction.^18^, ^19^ Still, the role of GC in treating KOA is unclear.

As a systematic pharmacological research method, network pharmacology has many similarities with the core idea of the holistic view of traditional Chinese medicine.^20^, ^21^, ^22^ Based on network pharmacology, the mechanisms and possible active ingredients of many TCMs against complex diseases were revealed. In this study, ultra‐performance liquid chromatography and quadrupole/time‐of‐flight mass spectrometry (UPLC‐Q‐TOF/MS) analysis was used to distinguish the main active substances from GC. Besides, the therapeutic effect of GC on KOA was studied in animal experiments. Finally, network pharmacology, molecular docking and validation studies were used to mining potential mechanisms of GC against KOA.

## 2 MATERIALS AND METHODS

### 2.1 Preparation of EC

*Glycyrrhiza uralensis* Fisch. was purchased from Zhejiang Chinese Medical University Chinese Herbal Pieces Co., Ltd. (Hangzhou, China) (lot. no. 210201). According to the dosage specified in the pharmacopoeia, GC extraction solution was prepared by hot reflux extraction method and stored in aliquots at −20°C until use. For UPLC‐Q‐TOF/MS analysis of GC, the extraction solution was diluted with methanol to 10 mg/mL and centrifuged (4°C, 12,000 rpm, and 10 min) to obtain the supernatant. The supernatant was filtered through a microporous membrane filter of 0.22 μm in diameter. Prior to analysis, the sample was stored at 4°C.

### 2.2 Analytical conditions of UPLC‐Q‐TOF/MS

UPLC system equipped with Waters C18 (2.1 mm × 100 mm, 1.7 μm particle size), and it was operated under the condition of 35°C column temperature, was utilized to separate the components. The mobile phases of gradient elution were acetonitrile (solvent A) and 1% formic acid solution (solvent B), respectively. At the flow rate of 0.30 mL/min, the elution process was 95% B from 0 to 2 min; 95%–0% B from 2 to 33.5 min; 0% B from 33.5 to 35 min. The injection volume was 1.0 μL for each running process. All the parameter conditions for mass spectrometry are presented in Table 1.

**TABLE 1:** Parameter conditions for mass spectrometry.

Mass spectrum conditions	
Scan range	m/z 50–1200
Electrospray ionization source	ESI
Temperature of electrospray ionization	120°C
Capillary voltages	3.0 kV
Cone well voltages	20 V
Argon flow rate	0.15 mL/min
Calibration standard	leucine‐enkephalin ([M–H]^−^ = 554.2615)

### 2.3 Animal experiments

Ten‐week‐old C57BL/6 male mice purchased from Hangzhou Medical College (Certificate number: SCXK (Zhe) 2019‐0002). The medial meniscus tibial ligament of C57BL/6 mice was made unstable during destabilization of the medial meniscus (DMM) surgery.^23^ All the mice were randomly divided into five groups: 1. Sham group, 2. DMM group, 3. GC low group, 4. GC medium group and 5. GC high group, and the ratio of high‐, medium‐ and low‐dose groups is 4:2:1. The mice in the Sham group and the DMM group were given normal saline orally. According to the animal dose conversion table, mice in GC high group received E.G treatment at a dose of 1.17 g/kg/day orally for 12 consecutive weeks. The Animal Care and Ethics Committee of Zhejiang Chinese Medical University approved all animal experiments and as per regulations of the Chinese Ministry of Science and Technology.

### 2.4 Behavioural analysis

Before collecting the samples, a DigGait Imaging System (Mouse Specifics, Boston, MA, USA) was utilized to follow and analyse the mice's gait. A high‐speed camera was used to capture the mice on a transparent treadmill measuring 18 cm/second. Stride length (cm), Stance (S), Stance Width (cm) and swing (s) were calculated to analyse the strides of the right hind. Pain levels in mice were measured using the hot plate method at 50°C. Recorded the time interval from reaching the platform surface to the start of the reaction.

### 2.5 Micro‐CT analysis

Twelve weeks after the operation, the right knee joints of the mice were taken and fixed with 4% paraformaldehyde for 3 days. The region of interest was identified as the area between the proximal tibia growth plate and the tibial plateau. Micro‐CT analysis yielded parameters such as percent bone volume (BV/TV, %), trabecular thickness (Tb.Th, mm) and bone mineral density (BMD, g/cm^3^).

### 2.6 Histopathology and immunohistochemistry analysis

Following the completion of micro‐CT scanning, the specimens were preserved in a 70% ethanol solution. Subsequently, they underwent decalcification in a 14% EDTA solution for a period of 14 days, culminating in paraffin embedding. Afterwards, sections with a thickness of 4 μm from the medial compartment of the joints were prepared for Alcian Blue Haematoxylin/Orange G and Toluidine Blue staining, aiming to assess the overall structural changes in the cartilage. Histomorphometric analysis was conducted using OsteoMeasure software (Decatur, GA). Three blinded observers scored the cartilage structure degeneration based on the guidelines provided by the Osteoarthritis Research Society International (OARSI). The expression of Collagen TypeII (Col2), matrix metalloproteinase 13 (MMP13) was observed by immunohistochemistry. Anti‐Col2 (Abcam, ab34712, 1:200), anti‐Aggrecan (Bioss, bs‐11655R, 1:200) and anti‐MMP13 (Abcam, ab39012, 1:100) were utilized in this study.

### 2.7 Network pharmacological analysis

TCMSP database was searched for GC compounds with oral bioavailability (OB) of 30% and drug‐likeness (DL) of 0.18. To identify potential target proteins, we searched the Uniprot database for “Homo sapiens” and “Reviewed,” respectively. The keyword “Knee Osteoarthritis” was imported into GeneCards, OMIM, PharmGkb, TTD and DrugBank to obtain targets related to Knee Osteoarthritis and remove duplicates. The above‐mentioned pooled targets were converted into formalized gene symbols through the UniProt database, and a component target network for drugs was constructed using Cytoscape 3.8.0 software. GC against KOA may target bioactive compounds and KOA as common targets. These common targets were uploaded to the PPI network through the STRING website. The confidence score was over 0.96 and it was limited to “Homo sapiens.”

Gene Ontology (GO) and Kyoto Encyclopedia of Genes and Genomes (KEGG) enrichment analyses were performed on the identified common targets using the DAVID database. GO analysis and KEGG analysis showed that biological processes (BPs), and signalling pathways were statistically significant (*p* < 0.05).

### 2.8 Molecular docking

Retrieve the molecular structure of a compound from the PubChem database and reconstructed in the ChemBio3D Ultra(14.0.0.117). The protein PDB IDS obtained from the RSCB protein database were JUN(1A02), MAPK3(3FHR), MAPK1(4FUX), AKT1(1UNP), TP53(6MXY), RELA(3QXY) and STAT3(6NJS). The receptors and ligands format is converted to pdbqt by AutoDockTools 1.5.6. The structure was optimized by removing water molecules and adding hydrogen atoms. Molecular docking studies were then performed using Autodock Vina.

### 2.9 Statistical analysis

Data were expressed as mean ± SD. Statistical difference between groups was evaluated using a one‐way analysis of variance followed by Student's *t*‐test. Differences between means were considered statistically significant at *p* < 0.05. Statistical analysis was performed using SPSS 25.0 statistical software.

## 3 RESULTS

### 3.1 Identification of ingredients in GC

**FIGURE 1:**
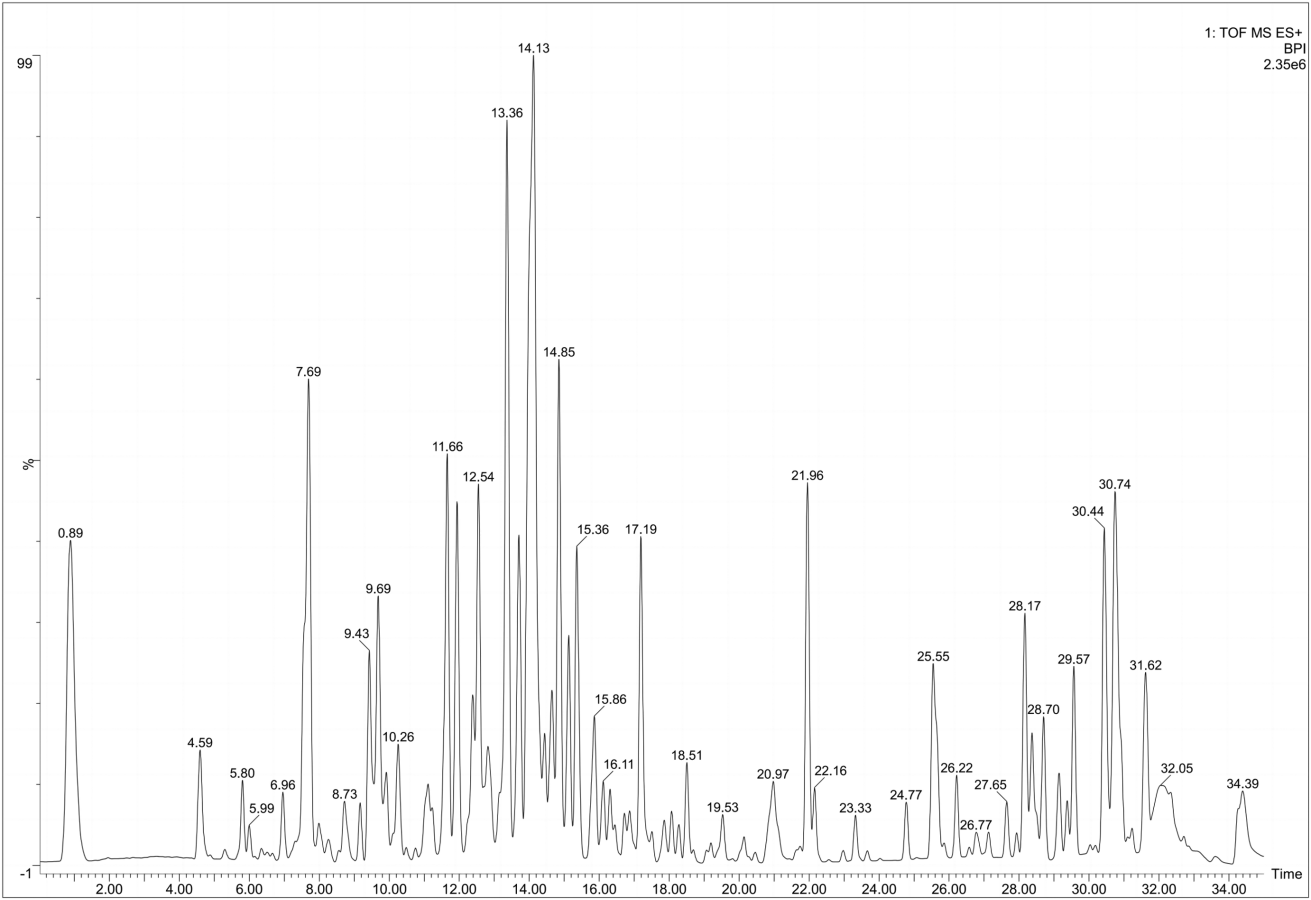
The total ion chromatogram of *Glycyrrhiza uralensis* Fisch. in positive ion modes.

Active ingredients were reliable thanks to UPLC‐Q‐TOF/MS analysis. As shown in Figure 1, GC was chromatographed using total ion chromatography in positive ion mode. Initial screening identified 15 compounds by comparing retention times, response values and published literature with reference standards. Flavonoids include Naringin, Schaftoside, Naringenin, Liquiritin apioside, Liquiritin, Glabrolide, Semilicoisoflavone B and Glyasperin A. Triterpenoids include Liquoric acid and beta‐Glycyrrhetinic acid. This finding confirms the richness of GC (Table 2).

**TABLE 2:** The detailed information of active ingredients contained in GC.

Component name	Chemical formula	Observation retention time (min)	Molecular mass number (Da)	Observed m/z	Detector count	Response value	Additives
Naringin	C_27_H_32_O_14_	5.80	580.17921	581.1862	18,809	4896	+H, +Na, +K
Schaftoside	C_26_H_28_O_14_	6.96	564.14791	565.1551	359,435	236,559	+H, +Na, +K
Liquiritin apioside	C_26_H_30_O_13_	7.69	550.16864	551.1761	332,013	144,028	+H, +Na, +K
Naringenin	C_15_H_12_O_5_	8.81	272.06847	273.0746	70,937	58,517	+H
Liquiritin	C_21_H_22_O_9_	9.43	418.12638	419.1335	380,230	288,193	+H
Liquoric acid	C_30_H_44_O_5_	11.66	484.31887	485.3255	256,263	145,208	+H
Glabrolide	C_30_H_44_O_4_	13.37	468.32396	469.3306	1,012,659	697,071	+H
Beta‐Glycyrrhetinic acid	C_30_H_46_O_4_	14.13	470.33961	471.3464	4,387,855	505,369	+H
Licorice‐saponin K2	C_42_H_62_O_16_	14.85	822.40379	823.4127	6,199,610	1,677,190	+H, +Na, +K
Licorice‐saponin C2	C_42_H_62_O_15_	15.36	806.40887	807.4152	202,199	6672	+H
Glepidotin B	C_20_H_20_O_5_	16.11	340.13107	341.1369	8027	4876	+H
Liconeolignan	C_21_H_22_O_5_	17.18	354.14672	355.1523	4132	4132	+H
Semilicoisoflavone B	C_20_H_16_O_6_	18.51	352.09469	353.1018	345,760	273,646	+H, +Na
Glyasperin A	C_25_H_26_O_6_	20.97	422.17294	423.1798	243,721	179,683	+H, +Na, +K
Gancaonin Q	C_25_H_26_O_5_	21.95	406.17802	407.1849	23,256	17,681	+H

### 3.2 GC improved dysmobility and pain in DMM‐induced KOA mice

**FIGURE 2:**
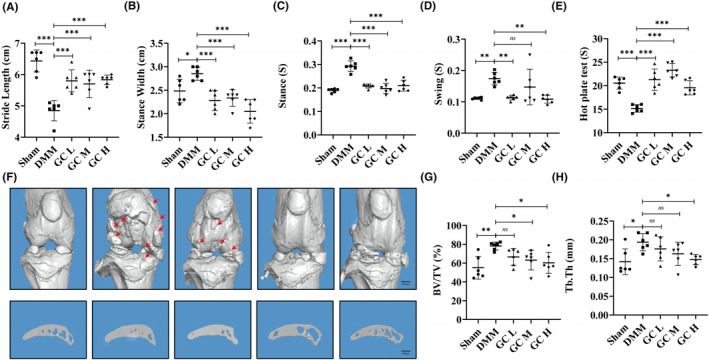
*Glycyrrhiza uralensis* Fisch. improved dysmobility and pain in DMM‐induced KOA mice. (A) Stride length (CM), (B) Stance Width (cm), (C) Stance (S), (D) Swing (S) of the right hind limb and (E) hot plate reaction time (S) of mice were detected. (F) Representative 3D reconstruction of the right knee joint and subchondral bone. Scale bar = 100 μm. (G) BV/TV (%) and (H) Tb.Th (mm) were quantitative analysis data of the subchondral bone. All data were taken as means ± standard deviations (*n* = 6). *p < 0.05, **p < 0.01, ***p < 0.001. BV, bone volume; DMM, destabilization of the medial meniscus; KOA, knee osteoarthritis; Tb.Th, trabecular thickness.

According to previous reports, the locomotor and analgesic activity of DMM‐induced KOA mice were altered by pain and knee joint dysfunction.^24^, ^25^ In this study, we used gait analysis and the hot plate test to determine whether GC could alleviate DMM‐induced symptoms in KOA mice. As shown in Figure 2A–E, compared with the sham group, mice in the DMM group had decreased Stride Length, and hot plate reaction time, and increased Stance Width, Stance time and Swing time. These gait parameters and pain thresholds were significantly improved after GC treatment.

### 3.3 GC improved pathological alterations in DMM‐induced osteoarthritic mice

**FIGURE 3:**
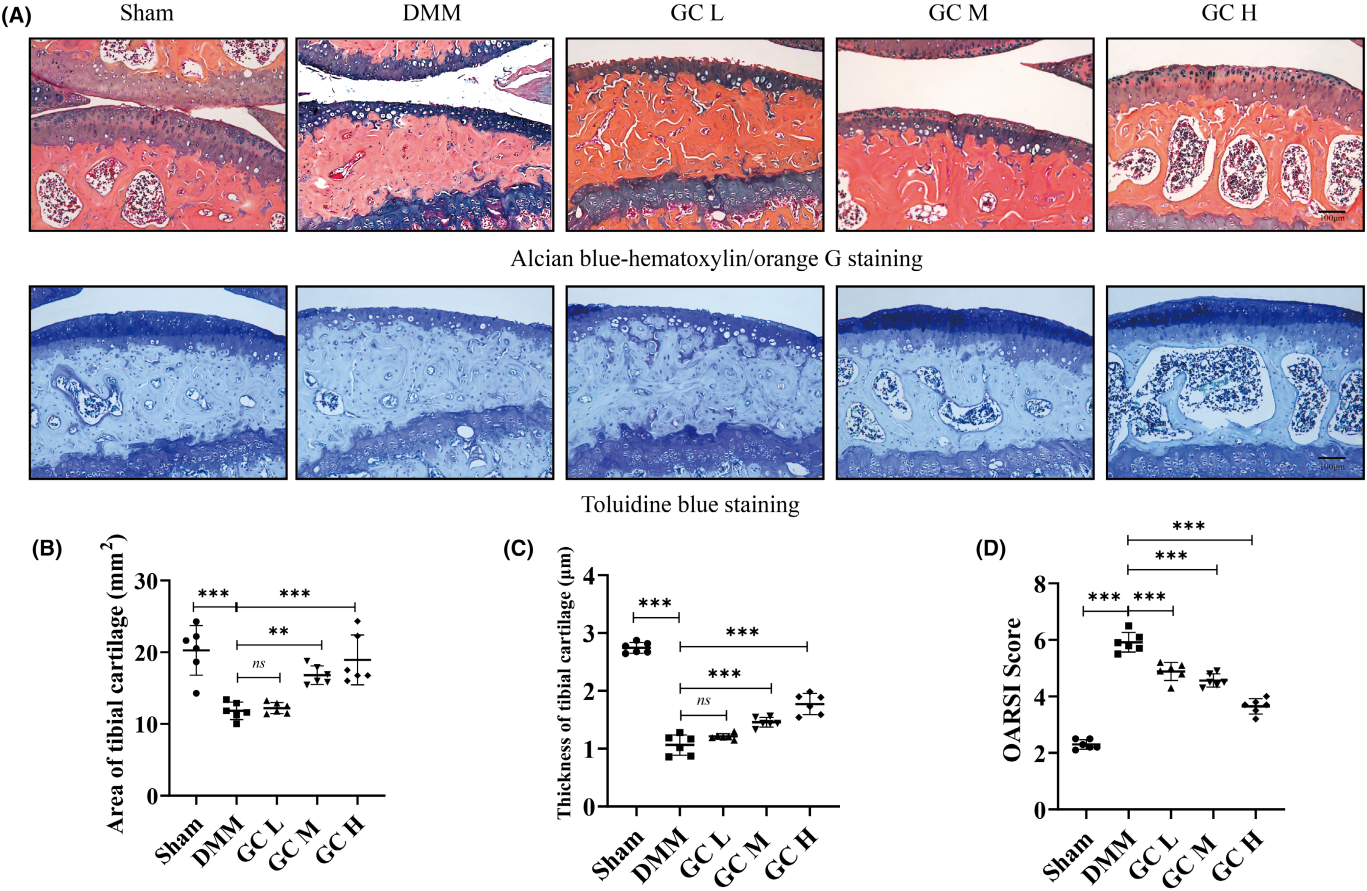
*Glycyrrhiza uralensis* Fisch. improved pathological features in DMM‐induced osteoarthritic mice. (A) ABH staining and TB staining of the right knee joint in C57BL/6 mice. Morphological quantitative analysis of (B) area of tibial cartilage (mm^2^) and (C) thickness of tibial cartilage (μm). (D) OARSI scoring of the sections analysed by histomorphometry. All data were taken as means ± standard deviations (*n* = 6). *p < 0.05, **p < 0.01, ***p < 0.001. DMM, destabilization of the medial meniscus; OARSI, Osteoarthritis Research Society International.

To investigate the therapeutic effect of GC on KOA, a mouse osteoarthritis model was established by DMM surgery. In DMM‐induced mice, articular cartilage was significantly damaged, with reduced cartilage area and thickness (Figure 3A–C). These histological findings were confirmed in the OARSI score of cartilage damage in mice, which was significantly higher in the DMM group than in the Sham group (Figure 3D). However, the cartilage tissue of the mice in the experimental group was relatively complete. In addition, subchondral bone microarchitecture and osteophyte formation were examined using μCT. Results showed that the number of subchondral osteophytes and the values of BV/TV and Tb.Th in the subchondral bone of DMM group were significantly higher than those in the sham group. After 12 weeks of GC treatment, osteophytes, BV/TV and Tb.Th in the subchondral bone were significantly suppressed compared with the DMM group (Figure 2F–H). In summary, GC could prevent articular cartilage impairment in osteoarthritic mice and reduce osteophytes formation.

### 3.4 GC promoted extracellular matrix synthesis in osteoarthritic articular cartilage

**FIGURE 4:**
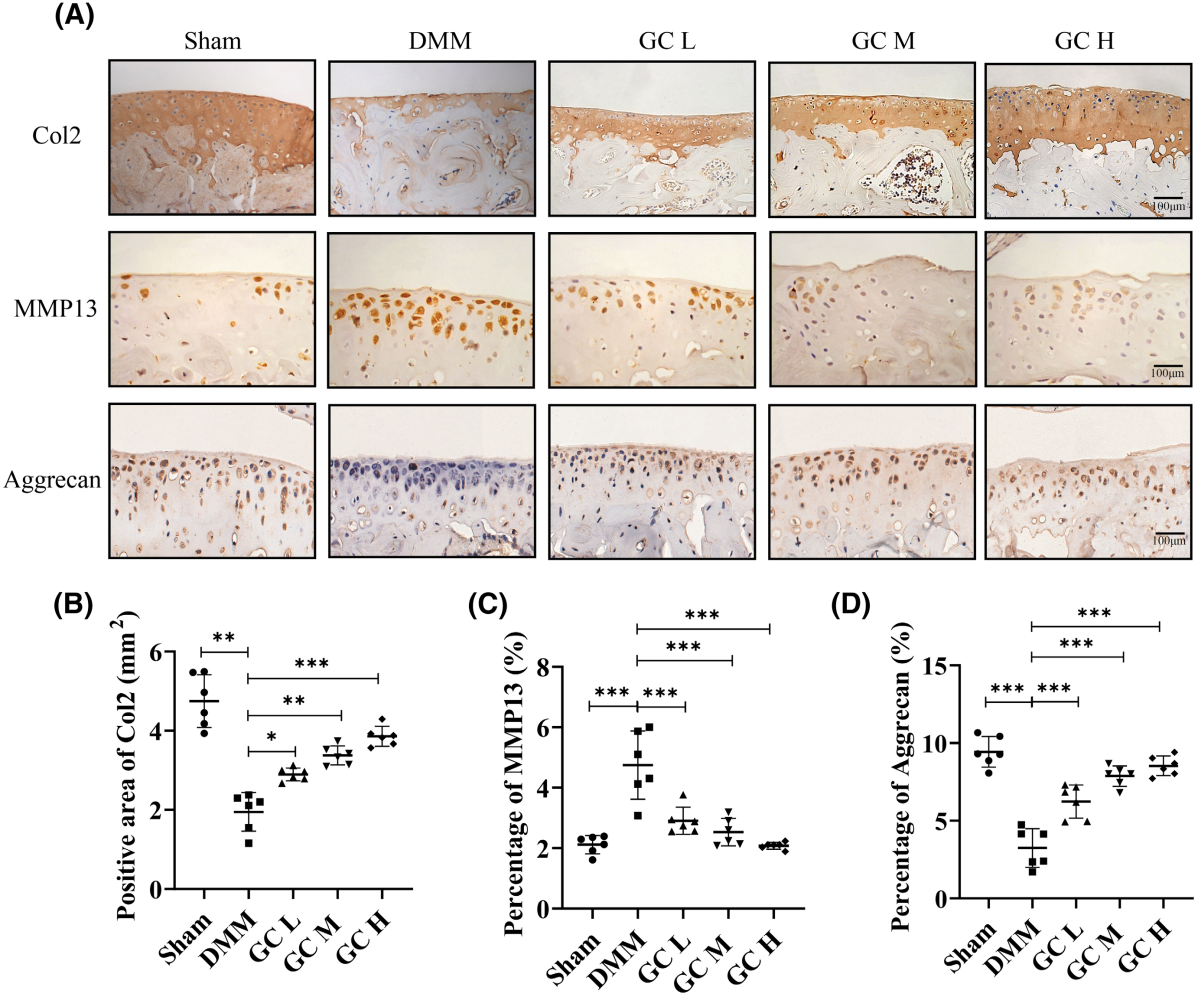
*Glycyrrhiza uralensis* Fisch. promoted extracellular matrix synthesis in osteoarthritic articular cartilage. (A) Immunohistochemical staining of MMP13, Col2 and Aggrecan in cartilage. Scale bar = 100 μm. (B–D) Quantification of the positive repression area of Col2 (mm^2^), percentage of positive expression of MMP13 and Aggrecan (%). All data were taken as means ± standard deviations (*n* = 6). *p < 0.05, **p <0.01, **p < 0.001.

In knee osteoarthritis, cartilage degeneration is primarily caused by an imbalance between anabolic and catabolic activity.^26^ Col2 and Aggrecan expression level was a representative marker of cartilage anabolic activity, and MMP13 was an important catabolic marker of knee osteoarthritis progression. In this study, immunohistochemical staining was used to evaluate whether GC could regulate the extracellular matrix metabolism of articular cartilage. Quantitative analysis showed that Col2 and Aggrecan were significantly downregulated and MMP13 was significantly upregulated in DMM‐induced KOA mice. GC reversed this trend by increasing Col2 and Aggrecan expression in cartilage and decreasing MMP13 levels in chondrocytes (Figure 4A–E). The above results proved that GC could protect cartilage from being degraded in vivo.

### 3.5 Identifying GC targets against KOA

**FIGURE 5:**
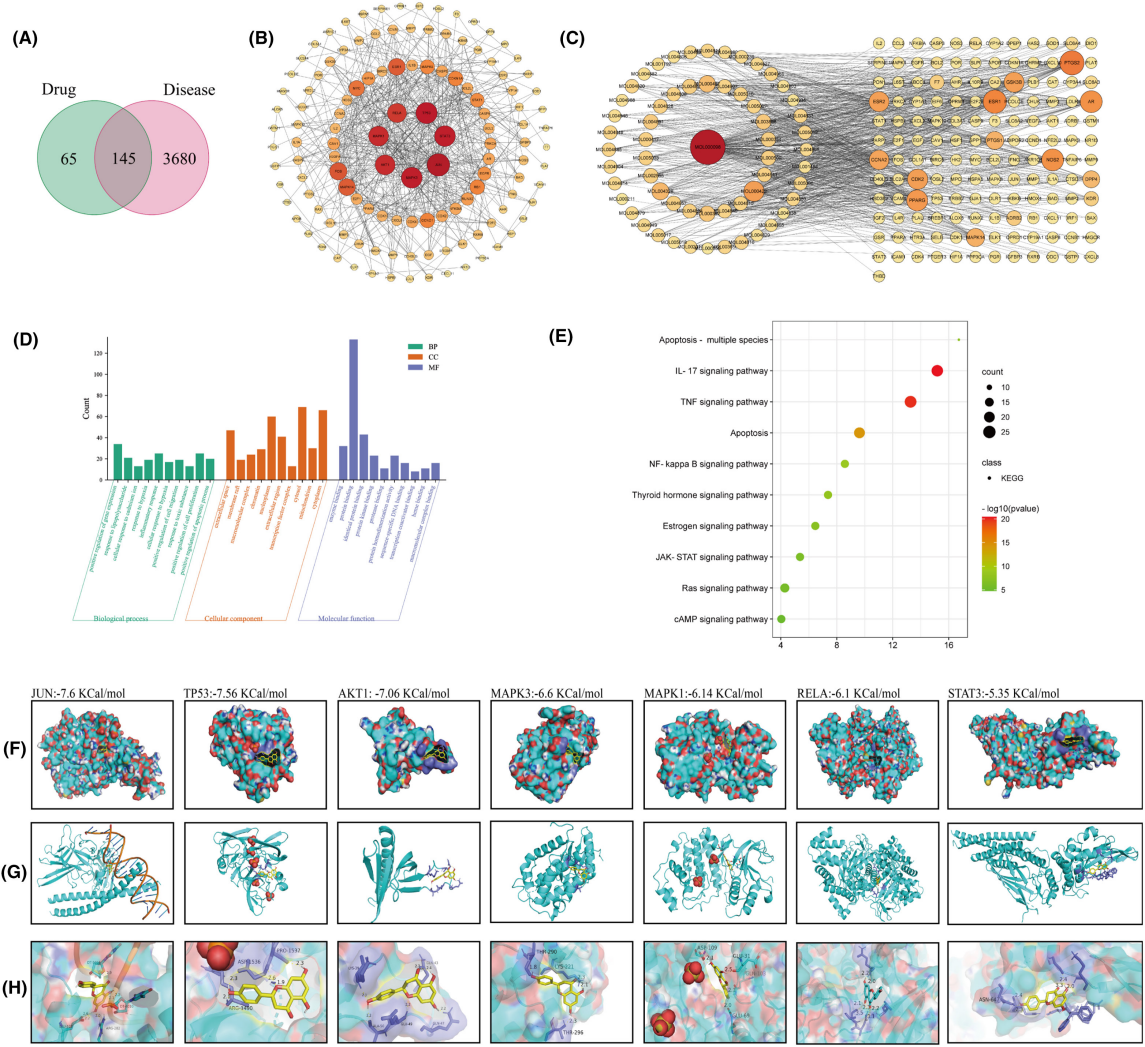
Network pharmacology analysis. (A) Overlaps between KOA's targets and GC drug targets, (B) PPI network visualization, (C) GC‐target‐KOA network. The circular node represents the traditional Chinese medicine component, and the rectangular node represents the disease target. The higher the correlation, the redder the colour, and vice versa, the greener the colour, (D) GO enrichment analysis and (E) KEGG pathways enrichment analysis. Molecular docking analysis. Molecular docking simulation results, including compounds, targets and minimum binding free energies, (F) surface appearances, (G) cartoon appearances of targets, (H) enlarged picture of the active site. GC, *Glycyrrhiza uralensis* Fisch.; GO, gene ontology; KEGG, Kyoto Encyclopedia of Genes and Genomes; KOA, knee osteoarthritis; PPI, protein–protein interaction.

The Traditional Chinese Medicine System Database and Analysis Platform (TCMSP) generated 210 drug targets after deduplication, while a total of 3825 KOA‐related targets were obtained from GeneCards, OMIM, PharmGkb, TTD and Drugbank databases. By comparing drug targets with KOA‐related targets, a total of 145 potential therapeutic targets were identified (Figure 5A). There were 124 nodes and 540 edges in the protein–protein interaction (PPI) network and the average node degree was 8.71 (Figure 5B). Top seven targets were JUN, MAPK3, MAPK1, AKT1, TP53, RELA and STAT3, suggesting that they have critical parts in the treatment of KOA.

As shown in Figure 5C, the network consists of 204 nodes (active ingredients and corresponding targets) and 719 edges (interaction relationship between active ingredients and target proteins). Among them, the circular nodes represent the ingredients of traditional Chinese medicine, the rectangular nodes represent the disease targets. The results showed that GC has great potential in the treatment of KOA. GO and KEGG results indicated that GC may treat KOA by positively regulating gene expression and inflammatory response through IL‐17, NF‐κB and TNF signalling pathways (Figure 5D,E).

### 3.6 Molecular docking

We further explored the relationship between active ingredients and the seven key protein targets. Molecular docking was used to analyse the binding energies of active ingredients with the seven key protein targets. We found the Naringenin had good binding affinities all with the seven key protein targets (Figure 5F–H). The hydrogen bonding energies of JUN, TP53, AKT1, MAPK3, MAPK1, RELA and STAT3 were −7.6, −7.56, −6.60, −6.14, −6.10 and −5.35 Kcal/mol, respectively. Although the molecular docking analysis indicated the binding ability of Naringenin with those seven protein targets, the regulation of GC on them in knee osteoarthritis still needs to be further investigated.

### 3.7 GC improved the inflammatory environment and inhibited the NF‐κB pathway in articular cartilage

**FIGURE 6:**
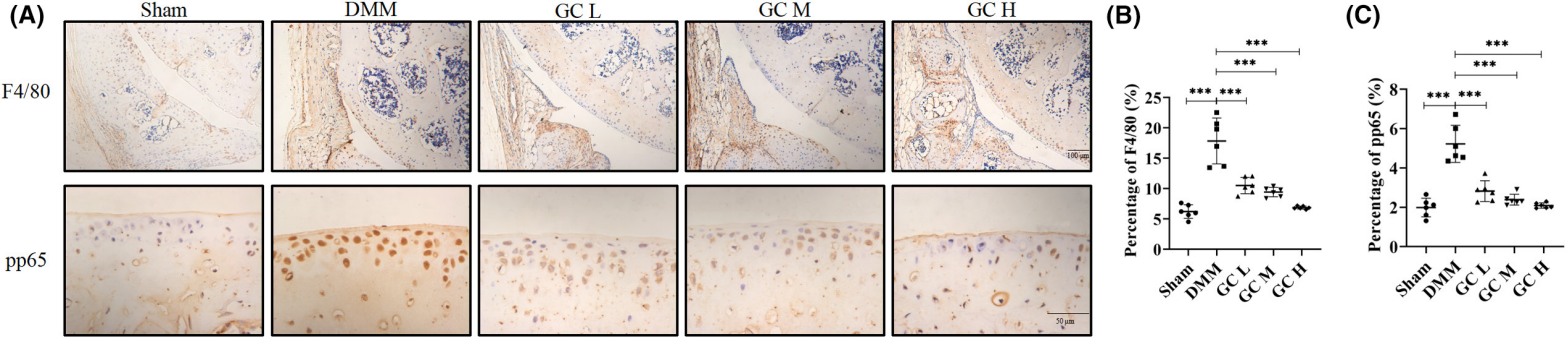
*Glycyrrhiza uralensis* Fisch. had anti‐inflammatory effects in DMM‐induced osteoarthritic mice. (A) Immunohistochemical staining of F4/80 and pp65. (B, C) Quantification of the percentage of positive expression of F4/80 and pp65 (%). All data were taken as means ± standard deviations (*n* = 6). *p < 0.05, **p < 0.01, ***p < 0.001. DMM, destabilization of the medial meniscus.

To further confirm whether GC had the anti‐inflammatory response in vivo, we observed the F4/80, a inflammatory marker. Result showed that GC could obviously inhibit the increased level of F4/80 in the synovium of DMM mice. Additionally, GO and KEGG analyses showed that the anti‐inflammatory ability of GC on KOA might concern with NF‐κB pathway. Immunohistochemical experiment was performed. Not surprisingly, the expression of pp65 was significantly upregulated in articular cartilage of DMM‐induced KOA mice, and GC could reverse the expression of pp65 to normal level (Figure 6). These results suggest that GC could play a significant role in inhibiting inflammation response, highly relating to its regulatory effect on NF‐κB pathway.

## 4 DISCUSSION

KOA is a degenerative orthopaedic condition that seriously affects the life quality of older people worldwide.^27^, ^28^ Apart from surgical treatment, few medications are effective in treating the symptoms of KOA, promoting cartilage regeneration, or preventing its degeneration. So far, the approved drugs for treating KOA are still very limited.^29^ Traditional Chinese Medicine (TCM) is becoming an indispensable option for KOA as it is plentiful in various active ingredients. So we wondered if there was a traditional Chinese herb that could improve the KOA process?

We detected the active ingredients contained in *Glycyrrhiza uralensis* (GC) using ultra‐performance liquid chromatography coupled to quadrupole time‐of‐flight mass spectrometry (UPLC‐Q‐TOF/MS). We found that GC is rich in flavonoids and triterpenoids, such as Naringin and Liquiritin apioside. GC became the first choice of clinical anti‐inflammatory and antibacterial drugs, which may be related to the existence of these two compounds. Limitation of knee joint motion and joint pain are the most obvious clinical manifestations in patients with knee osteoarthritis.^30^ The C57BL/6 mouse strain possesses consistent genetic backgrounds and relatively stable sensitivities to experimental drugs and treatments, which is conducive to assessing the impact of the various group treatments that we designed on the results. In the present study, 30 ten‐week‐old male C57BL/6 mice were used as animal samples. In animal experiments, we had indeed observed that GC could ameliorate the pathological changes caused by KOA. Excitingly, Our results showed that GC can improve the gait behaviour of DMM‐induced KOA mice, including Stride Length, Stance Width, Stance time, Swing time and hot plate reaction time (Figure 2). Meanwhile, long‐term mechanical load and inflammatory environment can lead to the excessive catabolic in articular cartilage, thereby promoting KOA formation.^31^ We observed the homeostasis of catabolic and anabolic effects of GC on the knee joint in vivo using immunohistochemical methods. GC could prevent the degradation of the extracellular matrix of articular cartilage by promoting the anabolic activity of articular cartilage (increasing Col2 level) and inhibiting the catabolic activity (decreasing MMP13 level) (Figure 4). Additionally, GC increased the area and thickness of articular cartilage in DMM‐induced KOA mice (Figure 3).

The network pharmacology approach is novel, integrating information from bioinformatics, systems biology and multiphasic pharmacology.^32^ TCM have complex components and interactions. Elucidating these interactions is critical for studying active ingredients and their mechanisms of action. Network pharmacology is a unique strategy to address these issues. Thus, we comprehensively analysed the relevant targets and potential pathways of GC using network pharmacology analysis. The results showed that GC was highly correlated with inflammation during the treatment of KOA. This was further established in the following molecular docking simulations. It is well known that inflammation is a key factor in the pathogenesis of osteoarthritis. Inflammatory environment leads to progressive inflammation of the joints and destruction of articular cartilage and bone. To verify this result, we examined inflammation‐related indicators. Synovial inflammation is an important pathogenic factor of KOA. Synovial macrophages can secrete a large number of soluble pro‐inflammatory factors to cause synovial inflammation. Previous studies have shown that F4/80 is a specific marker antibody on the surface of mature mouse macrophages. Therefore, we detected the expression of F4/80 in the synovium of the joint. The pp65 is a key regulatory protein in the NF‐κB signalling pathway, which itself has been shown to be involved in inflammatory signalling by numerous studies. Similarly, we examined the expression level of pp65 in articular cartilage. The results showed that F4/80 and pp65 were significantly upregulated in DMM‐induced KOA mice. GC reversed this trend by downregulating the expression of F4/80 in the synovium and pp65 in the articular cartilage. This means that GC was indeed slowing down the progress of KOA by suppressing inflammation. We also evaluated potential safety issues or side effects related to GC. As illustrated in Figure S1, the size and colour of kidneys and livers exhibited minimal variation across groups. HE staining revealed that the hepatocytes of mice were of normal size, well‐arranged in hepatic cords, and hepatic lobules were distinctly visible (Figure S2). As depicted in Figure S2, the morphology and dimensions of glomeruli in the DMM group were comparable to those in the Sham group, with uniform distribution. Based on these histopathological observations, it is plausible that GC has negligible hepatotoxicity or nephrotoxicity, as normal hepatic and renal architecture were retained in the treated group. In summary, GC is a medicinal herb that contains an abundant repertoire of bioactive constituents with demonstrated anti‐inflammatory efficacy and negligible side effects. GC exerts its anti‐inflammatory effects possibly through modulating the NF‐κB signalling pathway. These findings provide a scientific rationale for the traditional use of GC in inflammatory conditions.

To sum up, while identification of constituents by UPLC‐Q‐TOF/MS provides valuable chemical information on the compounds present in the extract further studies are warranted to rigorously evaluate the potential therapeutic relevance and mechanisms of action of these detected analytes. Quantitative analysis should be performed to ascertain the relative abundance of major constituents and guide subsequent bioactivity‐directed isolation work. Following purification of single compounds, their intrinsic biological activities could be assessed in vitro and in vivo and compared to the effects of the crude extract to discern the principal active principles. Moreover, exploring the known pharmacological and biological profiles of the identified compounds may shed light on plausible mechanisms, with reference made to relevant literature reports. Elucidating structure–activity relationships in suitable model systems could help establish structure–function correlations and prioritize candidates for further preclinical investigation. This also represents a limitation of the present study, in that it delineates a key direction for future work.

## AUTHOR CONTRIBUTIONS

**Jianbo Xu:** Conceptualization (lead); data curation (lead); methodology (lead); software (lead); writing – original draft (lead). **Qi Sun:** Conceptualization (equal); data curation (equal); methodology (equal); writing – review and editing (equal). **Min Qiu:** Data curation (equal); methodology (equal); software (equal). **Yungang Wu:** Conceptualization (equal); data curation (equal). **Liangyan Cheng:** Data curation (equal); validation (equal). **Nanwan Jiang:** Data curation (equal); validation (equal). **Ruogu Zhang:** Data curation (equal); methodology (equal); validation (equal). **Jiali Chen:** Conceptualization (equal); methodology (equal); supervision (equal). **Wenhua Yuan:** Data curation (equal); validation (equal). **Hongting Jin:** Conceptualization (equal); funding acquisition (equal); supervision (equal); writing – review and editing (equal). **Weidong Wang:** Conceptualization (equal); supervision (equal). **Yunhuo Cai:** Conceptualization (equal); supervision (equal). **Chunchun Zhang:** Conceptualization (equal); supervision (equal); writing – review and editing (equal). **Pinger Wang:** Conceptualization (equal); funding acquisition (equal); methodology (equal); supervision (equal); writing – review and editing (equal).

## FUNDING INFORMATION

This study was partially supported by Natural Science Foundation of China (grant nos. 82274550, 82074457 and 82104891), Zhejiang Provincial Natural Science Foundation of China (grant no. LY22H270005), Zhejiang Provincial Natural Science Fundfor Distinguished Young Scholar of China (grant no. LR23H270001), the Research Project of Zhejiang Chinese Medical University (grant no. 2022JKZKTS32) and the State Administration of Traditional Chinese Medicine of Zhejiang Province (grant nos. 2021ZZ014 and 2024ZL052).

## CONFLICT OF INTEREST STATEMENT

The authors have declared that no conflict of interest exists.

## Supporting information


Figure S1.

## Data Availability

The data that support the findings of this study are available from the corresponding author upon reasonable request.
