# Supplementary material for: Exploring the pharmacological mechanism of Glycyrrhiza uralensis against KOA through integrating network pharmacology and experimental assessment
Source: J Cell Mol Med. 2024 May 14;28(9):e18319. doi: 10.1111/jcmm.18319 (PMC11092526; doi:10.1111/jcmm.18319)
Supplement: Supplementary file 1 — Figure S1. [file JCMM-28-e18319-s001.docx]

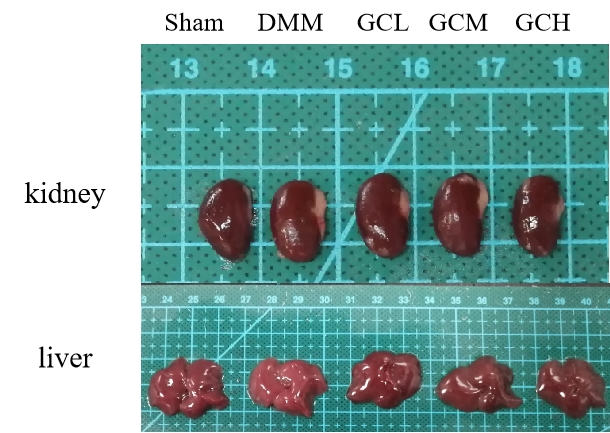


Fig. S1. Gross macroscopic view of mouse kidneys and livers.

Fig. S2. H&E staining of the livers and kidneys in C57BL/6 mice.
